# Novel Concepts for the Role of Chloride Cotransporters in Refractory Seizures

**DOI:** 10.14336/AD.2021.0129

**Published:** 2021-07-01

**Authors:** Pavel A Kipnis, Shilpa D Kadam

**Affiliations:** ^1^Neuroscience Laboratory, Hugo Moser Research Institute at Kennedy Krieger, Baltimore, MD 21205, USA.; ^2^Department of Neurology, Johns Hopkins University School of Medicine, Baltimore, MD 21205, USA.

**Keywords:** refractory seizures, TLE, HIE, TrkB, BDNF, KCC2, NKCC1

## Abstract

Epilepsy is associated with a multitude of acquired or genetic neurological disorders characterized by a predisposition to spontaneous recurrent seizures. An estimated 15 million patients worldwide have ongoing seizures despite optimal management and are classified as having refractory epilepsy. Early-life seizures like those caused by perinatal hypoxic ischemic encephalopathy (HIE) remain a clinical challenge because although transient, they are difficult to treat and associated with poor neurological outcomes. Pediatric epilepsy syndromes are consistently associated with intellectual disability and neurocognitive comorbidities. HIE and arterial ischemic stroke are the most common causes of seizures in term neonates and account for 7.5-20% of neonatal seizures. Standard first-line treatments such as phenobarbital (PB) and phenytoin fail to curb seizures in ~50% of neonates. In the long-term, HIE can result in hippocampal sclerosis and temporal lobe epilepsy (TLE), which is the most common adult epilepsy, ~30% of which is associated with refractory seizures. For patients with refractory TLE seizures, a viable option is the surgical resection of the epileptic foci. Novel insights gained from investigating the developmental role of Cl^-^ cotransporter function have helped to elucidate some of the mechanisms underlying the emergence of refractory seizures in both HIE and TLE. KCC2 as the chief Cl^-^ extruder in neurons is critical for enabling strong hyperpolarizing synaptic inhibition in the brain and has been implicated in the pathophysiology underlying both conditions. More recently, KCC2 function has become a novel therapeutic target to combat refractory seizures.

## Inhibition in the brain

Seizures are spontaneous hypersynchronous brain discharges that can last a few seconds to minutes and result from the neuronal circuit imbalance of excitation and inhibition. Early life acute onset seizures can have an underling etiology such as a genetic brain pathology that affect this E/I balance [1-3]. In contrast, epileptogenesis is the process by which a neuronal circuit undergoes permanent changes that increase the brain’s susceptibility to develop recurrent spontaneous seizures [3]. Epileptogenesis often follows an initial excitotoxic brain insult like early-life seizures of either acquired or genetic etiology, stroke, infection, or traumatic injury. Inherent seizure susceptibility is a group of factors such as age and background genetics [4-8] that influence the probability of the brain to throw a seizure given a similar insult. The two ages most susceptible for seizure occurrence during the human lifespan are early-life and geriatric [9, 10]. Currently used ASMs are not known to have any effect on either epileptogenesis or the inherent age-dependent susceptibility. The discovery of new paradigms to investigate and efficiently rescue epileptogenesis soon after the emergence of seizures is the goal to prevent the onset of chronic epilepsy and long-term comorbidities. Even a single seizure event can have long-term effects and valuable insights have been gained using preclinical rodent models [11, 12], many of which have focused on the role of GABAergic transmission in the CNS. This review will focus on the mechanisms that underlie seizure refractoriness in both HIE and TLE and provide an overview of current translational research focuses and proposed therapeutic paradigms.

## Factors controlling tonic vs phasic inhibition

GABARs consist of two major categories: GABA_A_Rs are ionotropic receptors, whereas GABA_B_Rs are a subset of G protein-coupled receptors (GPCRs) and are metabotropic. GABA_A_Rs are composed of five subunits, of which many subunit variants have been identified including six α, three β, three γ, one δ, three ρ, one π, one θ, and one ε [13]. The subunit composition of the GABA_A_Rs changes developmentally and serves variable roles, such as the ability of δ-containing GABA_A_Rs to detect extrasynaptic GABA thereby mediating persistent tonic inhibition [14, 15]. Tonic and phasic GABAR-mediated currents are essential for the regulation of different aspects of neurotransmission. Phasic GABAergic neurotransmission adapts rapidly to stimuli and is essential in synaptic integration through feed-forward inhibition, thereby ensuring that excitatory inputs are summed only when synchronized [16]. In contrast, tonic inhibition generally reduces the magnitude and duration of excitatory neurotransmission, thereby inhibiting excess and pathological neuronal excitability. Beyond their role as a mediator of inhibitory neurotransmission in the CNS, GABA_B_Rs have been shown to promote the secretion of BDNF through the activation of phospholipase C, as well as promote the maturation of GABAergic synapses in the rodent hippocampus [17]. Activation of presynaptic GABA_B_Rs in the mouse hippocampus has been shown to decrease synaptic vesicle release [18]. The diversity of GABAergic signaling demonstrates the importance of its acute regulation throughout development and why it is often highlighted as an underlying mechanism in many neurodevelopmental and neuropsychiatric pathologies.

Although excitatory GABAergic neurotransmission is essential for neuronal development in the neonatal brain, acute insult can lead to pathological and irreversible disruption of these circuits. 20-60% of incidents of epilepsy are a result of acute insults to the CNS [19]. Among these insults, HIE is one of the most common to result in acute neonatal seizures [20]. Despite the introduction of new antiseizure medications (ASMs) into the clinic, refractory seizure incidence in neonates remains unchanged [21]. Two first-line ASMs like phenobarbital (PB) and valproate have shown little evidence that they reduce the subsequent incidence of epilepsy, a result which is tied to the regulation of the transmembrane Cl^-^ gradient and the electroneutral ion transporters KCC2 and NKCC1.

Neonatal HIE can be devastating due to several mechanisms relating to postnatal neurogenesis [22]. GABA has been shown to regulate the maturation and migration of immature cortical neurons during development [23, 24], as well as adult-born granule cells [25]. The effect of disrupted GABAergic circuits in the brain may be particularly impactful on granule cell migration, as preclinical studies in rodent models have shown that a greater rate of granule cell neurogenesis in induced by seizures in the adult hippocampus [26]. As HIE is a leading cause of TLE later in life, these factors illustrate the importance and urgency of developing new ASMs that impede epileptogenesis in the developing circuits after acute early-life insults.

## Susceptibility to seizures

To stimulate synaptogenesis and development, excitation predominates over inhibition in the neonatal period and throughout early infancy [27, 28]. Glutamate is the primary excitatory neurotransmitter in the brain and great focus has been placed on the ionotropic glutamate receptors and their effects of the electrochemical gradients of Na^+^, K^+^, and Cl^-^ as they relate to development and seizure susceptibility. Ionotropic glutamate receptors are characterized into three subtypes: N-methyl-D-aspartate receptors (NMDARs), α-amino-3-hydroxy-5-methyl-4-isoxazole propionate receptors (AMPARs), and kainate receptors.

The subunit composition of these receptors is developmentally regulated [29] and is an underlying mechanism of differential neurotransmission in the developing and adult brains. AMPARs are variably composed of the subunits GluA1, GluA2, GluA3, and GluA4, which contain highly conserved ligand binding and transmembrane domains [30]. The precise subunit composition of these ionotropic receptors has important consequences on the electrochemical homeostasis of the developing cells. In AMPARs, GluA2 plays a critical role in Ca^2+^ permeability; AMPARs containing the GluA2 subunit will not permit Ca^2+^ influx. In rodents, there are fewer GluA2-containing receptors in the developing brain compared to the mature brain, which leads to greater Ca^2+^ influx and seizure susceptibility [31]. In P10-12 rats after hypoxia, GluA2 expression was lower in the hippocampus and neocortex [32].

## Preclinical Models for Epileptogenesis

The most commonly used preclinical models of epileptogenesis are post-status epilepticus (SE) models of TLE [33]; subcortical and temporal lesions are thought to drive ictogenesis in SE models [34]. Importantly, acute CNS insults frequently result in neuronal network reorganization which leads to epileptogenesis. One critical mechanism underlying these disruptions is the plasticity of GABAergic mechanisms in response to the insult. Commonly, it is suggested that epilepsy is a result of compromised GABAergic inhibition and/or recurrent excitation [35]. Compromised GABAergic inhibition results from loss of susceptible interneuron cell populations [36, 37] or compromised inhibition due to formation of new recurrent excitatory circuits [38]. Studies have demonstrated that certain populations of GABAergic cells are vulnerable in animal models of induced epilepsy [39]. Additionally, the GABAergic hypothesis of epileptogenesis suggests pathologies where GABAergic signaling results in depolarization instead of hyperpolarization; positive GABAR modulators have been shown to exacerbate seizures [40] instead of suppressing them [41], indicating that excitatory GABAergic neurotransmission can be ictogenic.

## Chloride Cotransporter Function in the CNS

GABAergic neurotransmission in the brain has been a focus of neuroscience research for many decades and has been shown to undergo a developmental switch from excitatory to inhibitory [42]. GABA functions as a neurotransmitter by activating the GABA_A_R and permitting Cl^-^ to passively flow across the open receptor according to its electrochemical transmembrane gradient. The direction of Cl^-^ flow is dependent on the intracellular chloride concentration ([Cl^-^]_i_) of the neuron. Neuronal [Cl^-^]_i_ is primarily controlled by two transporters at the plasma membrane: the primary neuronal Cl^-^ exporter KCC2 and Cl^-^ importer NKCC1. NKCC1 is encoded by the *SLC12A2* gene in humans and serves an essential role in the secondary active transport of Na^+^, K^+^, and Cl^-^ into neurons. The relative developmental expression patterns of NKCC1 and KCC2 are dynamic as expression of KCC2 increases during development; the increased ratio of KCC2 to NKCC1 [43] that stabilizes at 2yrs of age is thought to underlie the developmental switch of GABAergic signaling from excitatory to inhibitory. In the neonatal brain, NKCC1 is highly expressed and accumulates Cl^-^ intracellularly, whereas low KCC2 expression results in a lower rate of Cl^-^ extrusion. The greater rate of Cl^-^ import due to the lower KCC2 to NKCC1 ratio results in a higher [Cl^-^]_i_ in neonatal neurons compared to mature neurons. In immature cortical neurons which highly express NKCC1 but not KCC2, the activation of the GABA_A_R by GABA results in Cl^-^ efflux due to the higher [Cl^-^]_i_. Paradoxically, GABA_A_R activation in the neonatal brain results in depolarization of the cell instead of hyperpolarization due to Cl^-^ efflux and underlies the refractoriness associated with neonatal seizures [44, 45].

Two isoforms of NKCC1 have been studied extensively: NKCC1a and NKCC1b. Studies have shown that mRNA expression of the NKCC1a isoform occurs primarily in the brain [46]. NKCC1 activity has been shown to be stimulated by phosphorylation of residues Thr(203), Thr(207), and Thr(212) [47]. Inhibition of the WNK1 and WNK3 pathways have been shown to decrease NKCC1 phosphorylation and activity, thereby accelerating recovery after stroke [47] and reducing neuropathic pain [48]. Off-label use of the diuretic and NKCC1 antagonist bumetanide (BTN) was ineffective as an ASM [49]. An Nkcc1^-/-^ mouse model showed impaired balance and deafness but no evidence of seizures [50]. Recently, a study showed a loss of function mutation of *SLC12A2* in two sisters with encephalopathy and developmental abnormalities, but no seizures [51]. These studies suggest KCC2 plays a more critical role in ictogenesis than NKCC1.

Several isoforms of KCC2 have been identified in recent years, each with their own developmental expression patterns [52]. The most widely studied are the full-length isoforms KCC2a and KCC2b which differ in their N-termini [53]. In the gestational period and shortly after birth, KCC2a and KCC2b expression levels were similar in brainstem neurons in a mouse model, with KCC2b expression increasing with development [54]. Although the function of KCC2a is not fully understood [55], KCC2b is the predominant isoform in the CNS and functions as its primary neuronal Cl^-^ extruder.

The cotransporter activity of KCC2 is dependent on its presence at the plasma membrane which is controlled through post-translational phosphorylation and dephosphorylation [56]. The capacity for Cl^-^ extrusion by KCC2 is potentiated by phosphorylation of the residue serine 940 (S940) [57] which increases the cell surface stability of KCC2. Ca^2+^ influx from increased NMDAR activity has been shown to decrease S940 phosphorylation through protein phosphatase 1 [58], decreasing its plasma membrane stability and Cl^-^ extrusion capacity by promoting endocytosis. Another critical phosphorylation site of KCC2 is the residue threonine 1007 (T1007), which been shown to decrease membrane insertion [59, 60], thereby decreasing Cl^-^ extrusion capacity. These factors, along with the additional intracellular roles that KCC2 plays demonstrate the intricate spatiotemporal control exhibited on its function and expression during development.

**Figure 1.**
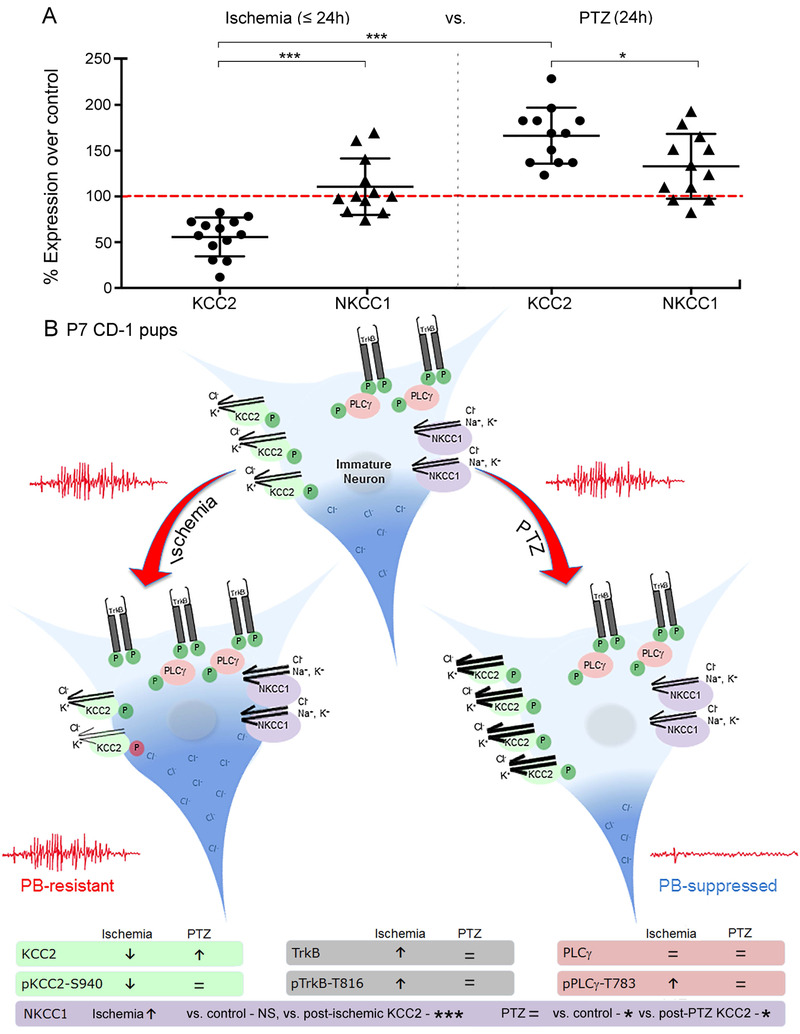
**Preclinical models of refractory vs. responsive neonatal seizures show differential effects on expression of KCC2. (A)** Significant differences in KCC2 expression between PTZ and ischemia models. **(B)** Schematic showing changes in KCC2 and TrkB pathways in PTZ and ischemia models. Figure 6 from [66].

Downregulation of KCC2 has been shown to be deleterious. In a *Kcc2*^-/-^ mouse model, KCC2 knockout mice showed early motor deficits which resulted in respiratory failure and death shortly after birth [61]. Aside from the effects of perturbed Cl^-^ homeostasis, KCC2 plays an essential role in the promotion of dendritic spine development through protein-protein interactions between the C-terminus of KCC2 and 4.1N [62], which mediates interactions between the cytoskeleton and plasma membrane. Spine formation is mediated by excitatory GABAergic neurotransmission during development [63], and aberrant spine morphology has been identified in multiple neuropsychiatric disorders including epilepsy and autism [64, 65]. These interwoven mechanisms highlight the delicate control of KCC2 expression throughout development and acknowledge the importance of rescuing post-insult KCC2 hypofunction. Interestingly, preclinical models have shown differences in KCC2 expression patterns after insult. After HIE, KCC2 expression decreases, whereas after treatment with the chemoconvulsant pentylenetetrazol (PTZ), KCC2 expression goes up [66] (Fig. 1A and 1B).

Recent discoveries related to the complex CNS functions of KCC2 have provided novel insights. Significant understanding has been gained into both the developmental switch of GABA-mediated depolarization to hyperpolarization during early development and in newborn neurons in the neurogenic niches in the adult brain as related to KCC2 expression and function. During development, the BDNF-mediated modulation in KCC2 expression [67] and BDNF-TrkB-mediated modulation of KCC2 membrane insertion and function [44, 68, 69] have initiated the understanding of a new role for KCC2 in the emergence of refractory seizures in neonatal brains where KCC2 hypofunction has been documented [45].

**Figure 2.**
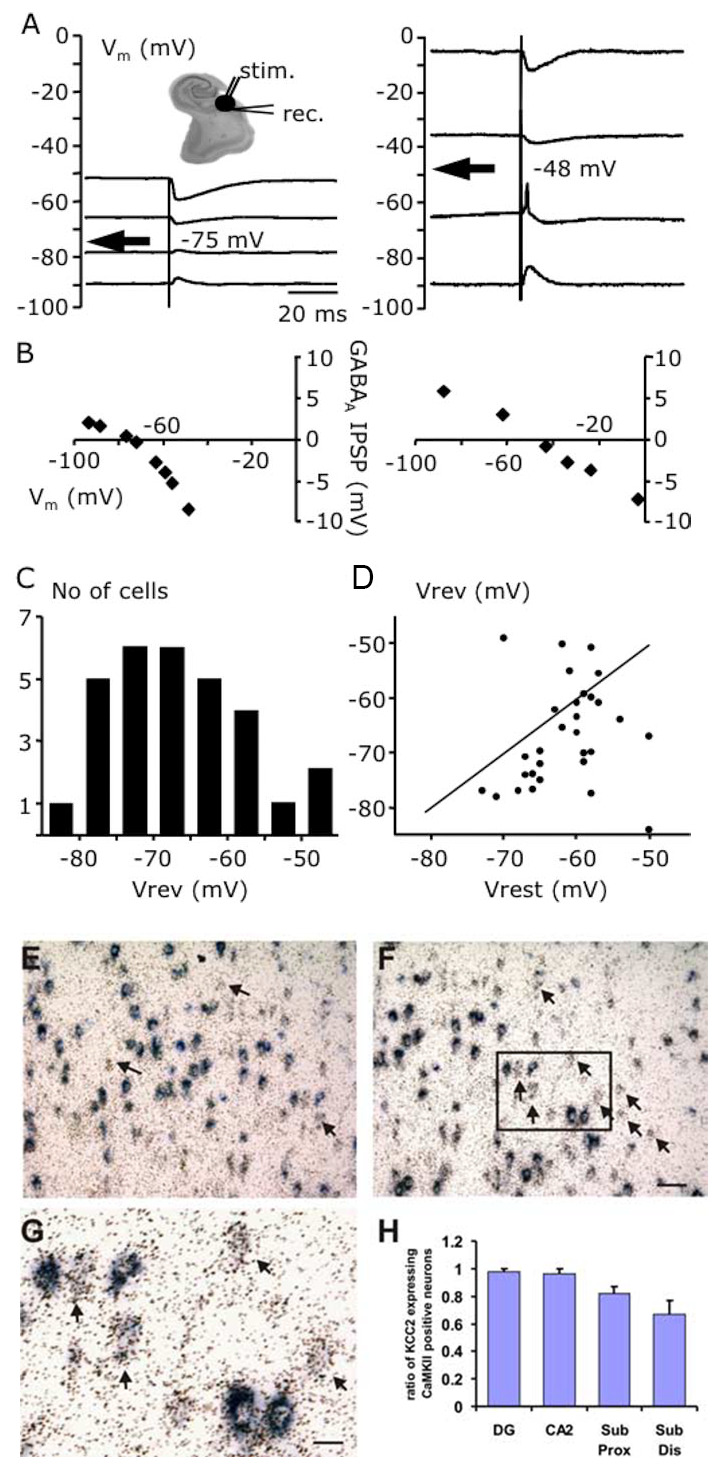
**Human mesial TLE patients showed depolarized reversal potential and lower KCC2 expression in surgically resected tissue. (A)** Values for V_rev_ for pyramidal cells of -75mV (left) and -48mV (right). **(B)** Synaptic events at varying V_m_. **(C)** Histogram showing V_rev_ for 30 pyramidal cells from subiculum. **(D)** Correlation of V_rev_ and V_rest_ for 30 subicular pyramidal cells. **(E, F)** Double *in situ* hybridization for KCC2 mRNA (BM purple) and CaMKIIα (S^35^) in the distal subiculum. **(G)** Expansion of black rectangle in F. (H) Percentage of CaMKIIα mRNA-positive cells that express KCC2 mRNA. Figure composed of Figures 2 and 3 taken from [70].

Rescuing KCC2 hypofunction through BDNF-TrkB pathway inactivation [44] or KCC2 functional enhancement [45] has been shown to rescue refractoriness in P7 CD-1 mice. In humans, KCC2 was downregulated in patients with refractory TLE (Fig. 2A-H) [70, 71]. *In vitro* studies performed on epileptic tissue gathered from patients with refractory TLE have shown lower levels of KCC2 mRNA in subicular pyramidal neurons (Fig. 2E-H) [70]. Rescuing NKCC1 hyperfunction using the NKCC1 antagonist and new generation short-acting diuretic bumetanide (BTN) has been effective in its off-label use in a wide range of neurological and psychiatric pathologies including Rett syndrome, autism, chronic pain, and epilepsy [63, 72]. These studies demonstrate that Cl^-^ homeostasis and the critical roles of KCC2 and NKCC1 underlie many of these neurological pathologies.

Phenobarbital is a positive allosteric modulator and agonist of the GABA_A_ receptor (GABA_A_R), thereby increasing GABA functional activity when administered [73]. This mechanism of action may underlie the documented inefficacy of PB against refractory neonatal seizures due to the expression patterns of KCC2 and NKCC1 [74]. Positive allosteric modulation of the GABA_A_R when GABA function is excitatory rather than inhibitory is a possible mechanism underlying the emergence of refractory acute neonatal seizures [44, 45, 69]. Downregulation of KCC2 has been demonstrated to increase neuronal excitability and epileptogenesis in rodent models [69], highlighting its recovery as a possible therapeutic paradigm [75].

Although off-label use of BTN has been shown to increase quality of life and alleviate symptoms in developmental disorders [49, 63], there has been growing concern that active efflux of BTN at the blood-brain barrier (BBB) by Oat3, Oatp1a4, and MRP4 is a limiting factor in the bioavailability of BTN in the brain [76]. In both invasive and non-invasive preclinical models of neonatal hypoxia, BTN failed to reduce seizure burden when given alone or concomitantly with PB [66, 68, 77, 78]. In a rat kindling model, BTN prevented increased kindling-induced seizure susceptibility at P11 but not at P14 or P21 [79]. A clinical trial that examined BTN efficacy as an anti-seizure agent in HIE reported its failure and significant side effects involving ototoxicity [80]. Another major concern surrounding BTN as a potential ASM is its short half-life as potent diuretic and antagonist of NKCC1/2. A BTN trial for HIE seizures conducted in the United States recently reported significant diuresis after a single dose but showed no definitive proof of its anti-seizure efficacy despite longer half-life of BTN [normal t_1/2_ = 1.5h] due to concomitant hypothermia protocols [81]. In contrast, recent clinical trials where BTN has been tested in the autism patient population have shown significant efficacy for rescuing the neuropsychiatric phenotype [82-87]. In two recent studies, a clinical trial for tuberous sclerosis complex patients with autism reported that BTN ameliorated neuropsychiatric behavioral problems but had no effect on seizure frequency or severity [86, 88], suggesting that NKCC1 antagonism with BTN is ineffective as a paradigm for reducing seizure burden.

To address the poor bioavailability of BTN due to the BBB, ester prodrugs of BTN have been developed and showed significantly higher brain penetrance than BTN, circumventing known obstacles involving ionization of BTN at physiological pH and tight binding of BTN to plasma proteins which underlie its poor brain penetrance [89] and low brain/plasma ratio. This has inspired the pursuit of novel BTN derivatives which selectively and potently inhibit NKCC1, although recent attempts have not been successful [90]. These factors confound NKCC1 as a target for the treatment of neonatal seizures and highlight the avenue of rescuing KCC2 hypofunction as a major focus of upcoming seizure and epilepsy research. Targeting ion cotransporters like NKCC1 and KCC2 carries impediments which include the temporal dynamics of plasma membrane protein stability, internationalization, protein turnover, and drug bioavailability. A thorough understanding of these factors will be essential in producing translationally viable research.

## Refractory Neonatal Seizures

The first two weeks of postnatal development in rodents are the rough equivalents of the first year in human postnatal development [42, 91] and this developmental window is the focus of many preclinical rodent models. Perinatal arterial ischemic stroke is the most common form of cerebral infarction in neonates, and results in symptoms such as neonatal seizures in 60% of incidents [92]. A rapid increase in endogenous BDNF following stroke have been reported [93-95]. Endogenous BDNF binding to the TrkB receptor has been shown to be responsible for the pathophysiological downregulation of KCC2 [96, 97]. K252a, a TrkB receptor antagonist, was shown to produce a significant increase in KCC2 mRNA and protein levels in organotypic hippocampal slice cultures [97]. In neonatal seizures the inefficacy of first-line anticonvulsant PB has been proposed to depend on the reversal of the Cl^-^ gradient which is maintained by KCC2. Age-dependent induction of BDNF after seizure activity may underlie the excessive release of BDNF in the immature brain. In a mouse model using unilateral carotid ligation without transection or infarct, it has recently been shown that the acute and transient post-stroke antagonism of the TrkB receptor may be a novel and effective way of curbing the emergence of pharmaco-resistant seizures in the neonatal brain [68]. This transient block also rescued the acute and sub-acute downregulation of KCC2 which otherwise could be detrimental in a maturing brain given the important role of KCC2 in dendritic spine formation, AMPA receptor traffic, and formation of functional GABA synapses [62]. Ischemia-related transient downregulation of KCC2 may also underlie the transient nature of HIE related neonatal seizures. ANA-12 is a recently identified low-molecular weight antagonist of TrkB [98]. It binds to the extracellular domain of TrkB, prevents BDNF-induced TrkB activation, and abolishes the biological effects of BDNF on TrkB-expressing cells but not those of NGF or NT-3 on TrkA and TrkC expressing cells. More importantly, ANA-12 inhibited TrkB function in the brain after systemic administration without any known adverse effects even with multiple doses [69]. Paradoxically, similar results were documented using the TrkB agonists LM22A-4, HIOC, and deoxygedunin, which rescued neonatal refractory seizures at P7 in CD-1 mice [44]. The manipulation of BDNF pathway-related microRNA (miRNA) expression may be a promising strategy to prevent epileptogenesis [99]. miR-155 is a miRNA that involved in a multitude of biological functions, including inflammatory responses in the CNS [100]. It has also been shown to repress *Bdnf* by binding to its 3'UTR [101], which makes miR-155 a possible therapeutic target for anti-epileptogenesis.

## Refractory TLE Seizures

In contrast to early-life refractory seizures associated with HIE, temporal lobe epilepsy (TLE) is an epilepsy syndrome associated with seizures predominantly initiated in the temporal lobe and is often accompanied by temporal lobe pathology like sclerosis, mossy fiber sprouting, and hippocampal degeneration [102-105]. The known causes for TLE are multi-pronged but often remain unknown [33, 106]. Brain injury, infection, tumors, malformations, inflammation are some of the known conditions that can precede TLE. ~40% of TLE patients become refractory significantly affecting the quality-of-life despite being on a combination of 2 or more ASMs at the highest daily dose regimens. This cohort of refractory patients generally end up opting for resective surgery to remove the seizure initiating focus which is usually the anterior horn of the hippocampus in the temporal lobe [107]. However, patients who did not become seizure-free after elective surgery have shown accelerated and progressive memory impairment [108]. Subiculum tissue resected from refractory TLE patients with hippocampal sclerosis showed spontaneous interictal-like activity *in vitro* which correlated negatively with KCC2 expression [70]. Similar studies have shown decreased KCC2 transcription and increased NKCC1 transcription in resected TLE tissue [109]. TLE has been broadly associated with impaired Cl^-^ homeostasis and depolarizing GABAergic transmission [110]. Similar to inhibition of the BDNF-TrkB pathway in rescuing neonatal HIE refractoriness, sequestration or inhibition of the TrkB-pathway in an adult model of temporal lobe epilepsy have shown promising results [111, 112].

One of the leading causes of TLE and hippocampal sclerosis is early-life seizures, especially febrile status epilepticus (FSE) [113, 114]. Mutations in *SLC12A5*, the gene which codes for KCC2, have been reported in patients with febrile seizures [115] which suggests that mutations in its coding or regulatory regions may contribute to the genetic susceptibility of TLE. Hippocampal sclerosis is the most common pathology underlying TLE [116] detected on MRI. Coronal imaging orthogonal to the long axis of the hippocampus reveals reduced hippocampal volume and perturbed hippocampal cytoarchitecture. Hippocampal sclerosis is often accompanied by mossy fiber sprouting [117] which is thought to underly hippocampal hyperexcitability. Patients with refractory mesial TLE whose quality of life is greatly perturbed can elect to undergo anteromesial temporal resection to remove the ictogenic tissue [118]. Much of what has been learned about TLE in the human brain is a result of *ex vivo* studies performed on resected tissue from refractory TLE patients. Although this data has provided a wealth of insight on the pathology, it confounds the ability to make a causal connection between TLE and its underlying mechanisms such as perturbed Cl^-^ homeostasis caused by KCC2 hypoexpression or hypofunction. However, much has been learned by studying the sclerosed hippocampi in preclinical models of TLE, which have shown similar pathophysiology like mossy fiber sprouting in the dorsal hippocampi [119]. Attempts to increase PB efficacy in a pilocarpine-induced epilepsy adult rodent model showed that the NKCC1 inhibitors BTN, azosemide, or torasemide did not improve PB efficacy [120], suggesting that NKCC1 antagonism is not a promising avenue for rescuing refractory epilepsies.

## The future of clinical trials aimed at antiepileptogenesis

**Figure 3.**
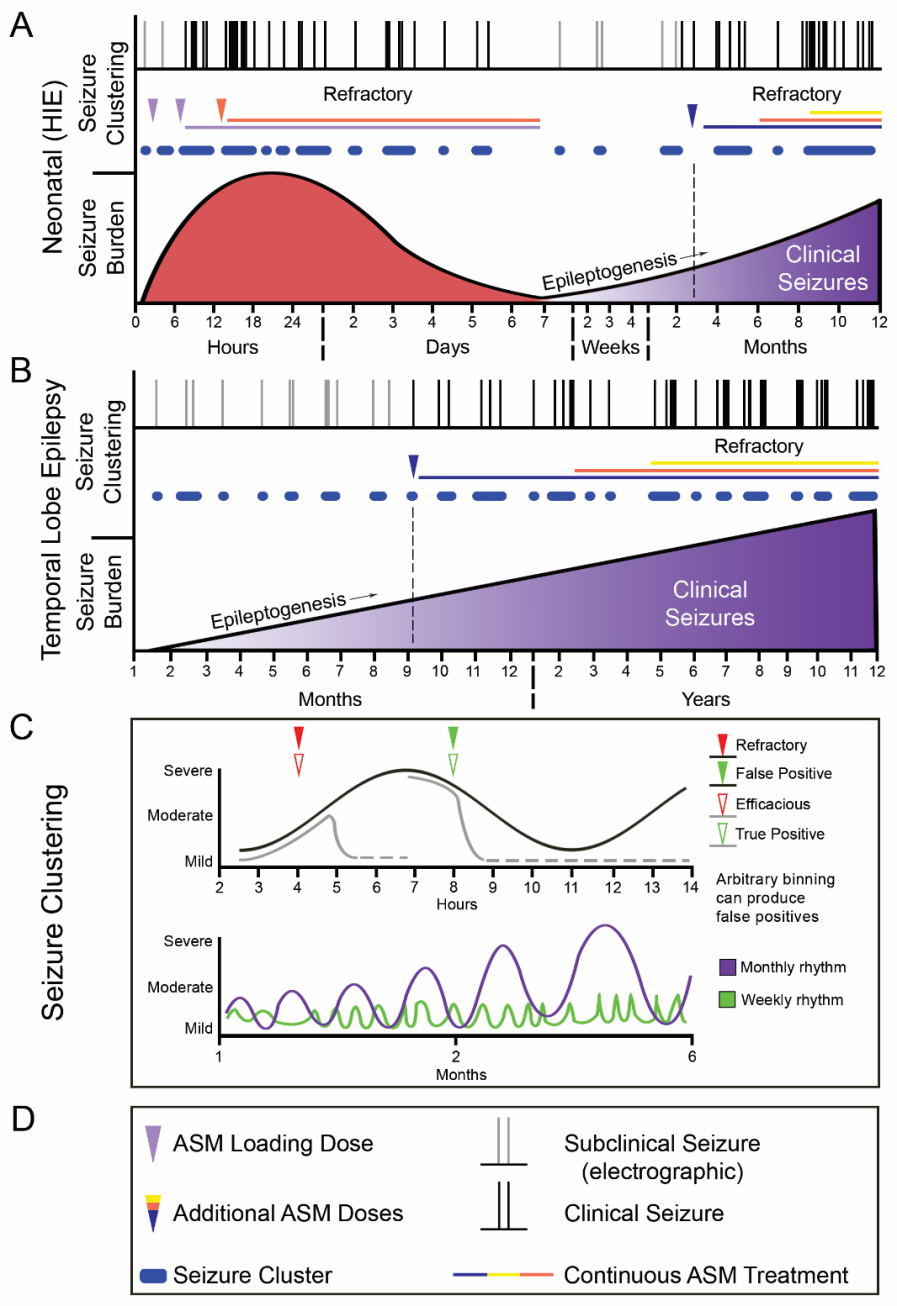
*Seizure burden and clustering in HIE and TLE related to emergence of refractory seizures. (A)* Seizure burden and clustering in HIE over the first few hours, days, weeks, and months of life. Seizures show clustering and average seizure burden decreases around day 3 [124]. Epileptogenesis following insult results in the emergence of clinical seizures in the following months. **(B)** Seizure burden and clustering in TLE over the months and years of the epileptogenic process. Electrographic seizures gradually develop into clinical seizures and exhibit increased clustering. **(C)** Expansion showing the peak-trough nature of refractory HIE seizures. Clinical interpretation of drug administration is dependent upon timepoint of drug administration and can demonstrate refractoriness or false positives (black line). Grey line demonstrates what efficacious drug administration would show on EEG. Green and purple curves show weekly and monthly seizure frequency rhythms [121], respectively. **(D)** Legend for graphical schematic.

Continuous EEG (cEEG) studies and clinical data have reported clustering of seizures in the acute and chronic phases of both HIE and TLE (Fig. 3A and 3B). Targeting KCC2 hypofunction as a mechanism underlying the emergence of refractory seizures relies on the ability for a long-term (months rather than hours) reversal; this requires taking seizure clustering into consideration. With the peak-trough variation in seizure frequency, clinical trials need to be wary of false positives due to administration of an ASM at a time point where seizure frequency is already declining naturally and regression to the mean is variable between patients (Fig. 3C). Granular plotting of temporal seizure frequency and clustering over a 5-day period from a hypothermia study indicate that variability of clustering is a prominent characteristic of HIE seizures. Reviewing time points when drug interventions occurred in the study in relation to the clustering phenotype of the seizures is critical because there is a high risk of false positives or false negatives when data for ASM efficacy are binned over short durations of a couple hours. A similar concept applies to chronic refractory epilepsy like TLE (Fig. 3B-D) where a few days of monitoring would fail to accurately detect both severity of the epilepsy at baseline and the efficacy or failure of an ASM.

The multi-day rhythms inherent in these pathologies [121], as well as the variable temporal patterns of seizure frequency between patients and variable baseline seizure burdens demonstrate the importance of appropriate statistical analysis and binning of data to show true efficacy of an intervention. An anti-seizure effect for HIE intervention would lower seizure burdens over 24h, which can only be reported through longer continuous EEG recording and binning durations that take into account the clustering nature of the ictal events. These insights will also permit the design of evidence-based guidelines for repeated dosing regimens since the goal is to significantly suppress all acute seizures in the first week of neonatal life. Similar strategies apply to targeting the anti-epileptogenic process for the evolution of TLE pathogenesis; long-term cEEG monitoring will help determine if ASM treatment paradigms contribute to the reversal of both the refractory epilepsy and its severity. Studies designed to identify the efficacy of novel anti-epileptogenic interventions would also need long-term cEEG using the latest invasive and non-invasive technology [121, 122] to identify sub-clinical and nocturnal seizure clusters. This will help prevent false positive and false negative results.

## Conclusion

**Figure 4.**
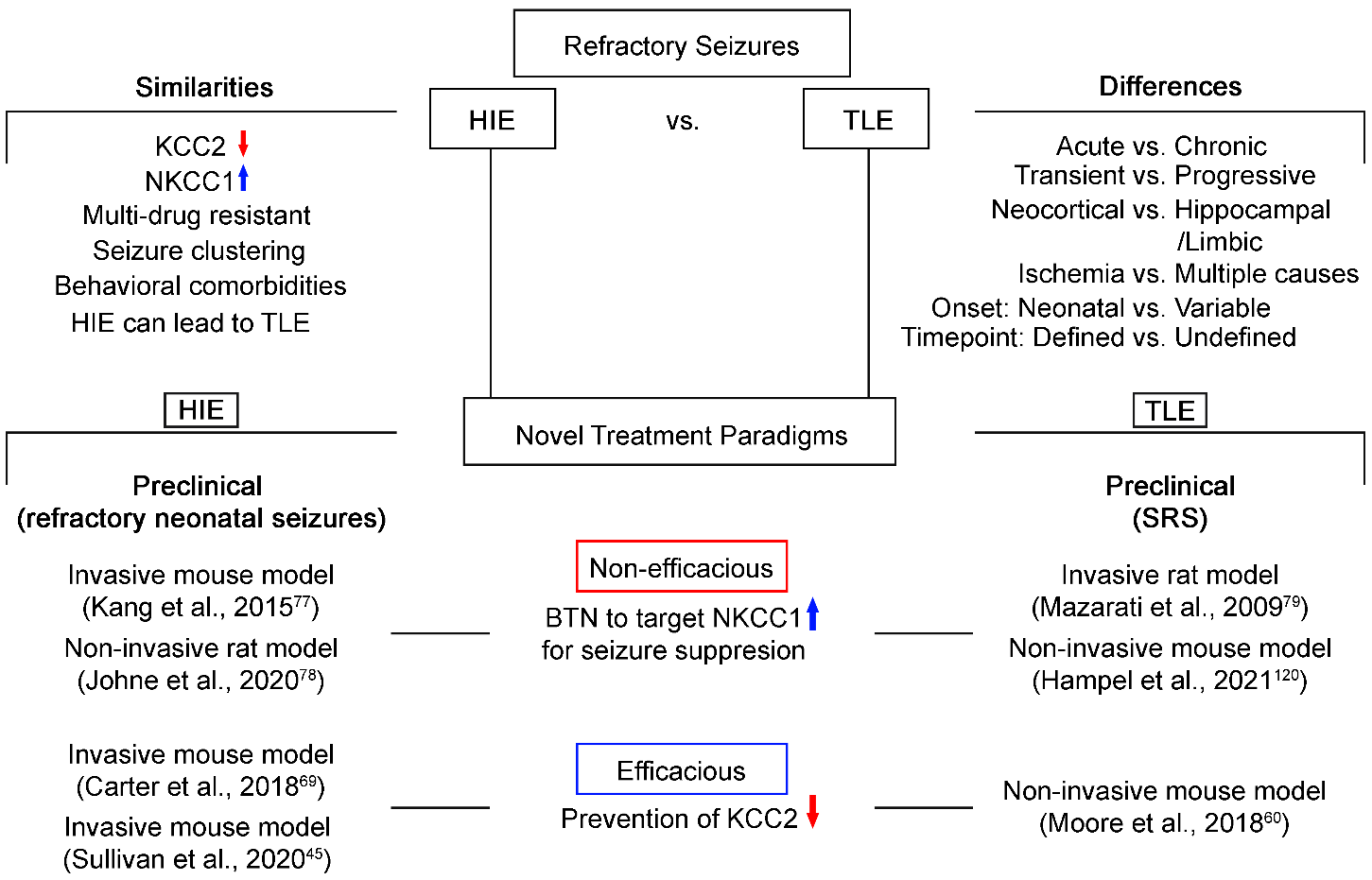
Schematic showing similarities and differences in HIE and TLE pathologies and treatment paradigms.

Although HIE is neonatal and transient and TLE is chronic and progressive, the underlying pathophysiology related to emergence of refractoriness and long-term comorbidities in affected patients overlap. Understanding the novel mechanisms that underlie the emergence of seizure refractoriness such as Cl^-^ cotransporter dysfunction can help to develop novel strategies for preventing refractoriness by modulating both seizure susceptibility and epileptogenesis. Preclinical research has shown great promise in manipulating KCC2 as a therapeutic target in rescuing refractoriness in HIE and TLE (Fig. 4). Manipulation of NKCC1 through diuretics like BTN and its analogues has been shown to be inefficacious in both preclinical research and clinical trials. Early intervention and prevention of epileptogenesis [123] remains an unmet need and is a focus of translational neuroscience research.

## References

[1] Scharfman HE (2007). The neurobiology of epilepsy. Curr Neurol Neurosci Rep, 7:348-354. 17618543 10.1007/s11910-007-0053-z PMC2492886

[2] Kang SK, Kadam SD (2015). Neonatal Seizures: Impact on Neurodevelopmental Outcomes. Frontiers in Pediatrics, 3:101. 26636052 10.3389/fped.2015.00101 PMC4655485

[3] Staley K (2015). Molecular mechanisms of epilepsy. Nat Neurosci, 18:367-372. 25710839 10.1038/nn.3947 PMC4409128

[4] Jensen FE (2006). Pediatric epilepsy models. Epilepsy Res, 68:28-31. 16377142 10.1016/j.eplepsyres.2005.09.013

[5] Auvin S, Pineda E, Shin D, Gressens P, Mazarati A (2012). Novel Animal Models of Pediatric Epilepsy. Neurotherapeutics, 9:245-261. 22467296 10.1007/s13311-012-0119-8 PMC3337021

[6] Pitkänen A, Engel J (2014). Past and Present Definitions of Epileptogenesis and Its Biomarkers. Neurotherapeutics, 11:231-241. 24492975 10.1007/s13311-014-0257-2 PMC3996117

[7] Glass HC, Shellhaas RA, Wusthoff CJ, Chang T, Abend NS, Chu CJ, et al (2016). Contemporary Profile of Seizures in Neonates: A Prospective Cohort Study. J Pediatr, 174:98-103.e1. 27106855 10.1016/j.jpeds.2016.03.035 PMC4925241

[8] Glass HC, Numis AL, Gano D, Bali V, Rogers EE (2018). Outcomes After Acute Symptomatic Seizures in Children Admitted to a Neonatal Neurocritical Care Service. Pediatr Neurol, 84:39-45. 29886041 10.1016/j.pediatrneurol.2018.03.016

[9] Liu S, Yu W, Lü Y (2016). The causes of new-onset epilepsy and seizures in the elderly. Neuropsychiatr Dis Treat, 12:1425-1434. 27382285 10.2147/NDT.S107905 PMC4918803

[10] Sen A, Jette N, Husain M, Sander JW (2020). Epilepsy in older people. Lancet, 395:735-748. 32113502 10.1016/S0140-6736(19)33064-8

[11] Fisher RS, Acevedo C, Arzimanoglou A, Bogacz A, Cross JH, Elger CE, et al (2014). ILAE official report: a practical clinical definition of epilepsy. Epilepsia, 55:475-482. 24730690 10.1111/epi.12550

[12] Löscher W (2017). Animal Models of Seizures and Epilepsy: Past, Present, and Future Role for the Discovery of Antiseizure Drugs. Neurochem Res, 42:1873-1888. 28290134 10.1007/s11064-017-2222-z

[13] Fritschy J-M, Panzanelli P (2014). GABAA receptors and plasticity of inhibitory neurotransmission in the central nervous system. Eur J Neurosci, 39:1845-1865. 24628861 10.1111/ejn.12534

[14] Walker MC, Semyanov A (2008). Regulation of excitability by extrasynaptic GABA(A) receptors. Results Probl Cell Differ, 44:29-48. 17671772 10.1007/400_2007_030

[15] Zheleznova NN, Sedelnikova A, Weiss DS (2009). Function and modulation of δ-containing GABAA receptors. Psychoneuroendocrinology, 34S1:S67-S73. 10.1016/j.psyneuen.2009.08.010 PMC2794972 19766404

[16] Contestabile A, Magara S, Cancedda L (2017). The GABAergic Hypothesis for Cognitive Disabilities in Down Syndrome. Front Cell Neurosci. doi: 10.3389/fncel.2017.00054. PMC5339239 28326014

[17] Kuczewski N, Fuchs C, Ferrand N, Jovanovic JN, Gaiarsa J-L, Porcher C (2011). Mechanism of GABAB receptor-induced BDNF secretion and promotion of GABAA receptor membrane expression. J Neurochem, 118:533-545. 21255015 10.1111/j.1471-4159.2011.07192.x

[18] Rost BR, Nicholson P, Ahnert-Hilger G, Rummel A, Rosenmund C, Breustedt J, et al (2011). Activation of metabotropic GABA receptors increases the energy barrier for vesicle fusion. J Cell Sci, 124:3066-3073. 21852427 10.1242/jcs.074963

[19] Banerjee PN, Filippi D, Hauser WA (2009). The descriptive epidemiology of epilepsy-a review. Epilepsy Res, 85:31-45. 19369037 10.1016/j.eplepsyres.2009.03.003 PMC2696575

[20] Volpe JJ (1981). Neurology of the newborn. Major Probl Clin Pediatr, 22:1-648. 7022034

[21] French Jacqueline A (2007). Refractory Epilepsy: Clinical Overview. Epilepsia, 48:3-7. 10.1111/j.1528-1167.2007.00992.x 17316406

[22] Kempermann G (2015). Adult Neurogenesis: An Evolutionary Perspective. Cold Spring Harb Perspect Biol, 8:a018986. 26684183 10.1101/cshperspect.a018986 PMC4743076

[23] Cuzon VC, Yeh PW, Cheng Q, Yeh HH (2006). Ambient GABA promotes cortical entry of tangentially migrating cells derived from the medial ganglionic eminence. Cereb Cortex, 16:1377-1388. 16339085 10.1093/cercor/bhj084

[24] Cancedda L (2007). Excitatory GABA action is essential for morphological maturation of cortical neurons in vivo. J Neurosci, 27:5224-5235. 17494709 10.1523/JNEUROSCI.5169-06.2007 PMC6672363

[25] Dieni CV, Chancey JH, Overstreet-Wadiche LS (2012). Dynamic functions of GABA signaling during granule cell maturation. Front Neural Circuits, 6:113. 23316139 10.3389/fncir.2012.00113 PMC3539683

[26] Parent JM, Yu TW, Leibowitz RT, Geschwind DH, Sloviter RS, Lowenstein DH (1997). Dentate granule cell neurogenesis is increased by seizures and contributes to aberrant network reorganization in the adult rat hippocampus. J Neurosci, 17:3727-3738. 9133393 10.1523/JNEUROSCI.17-10-03727.1997 PMC6573703

[27] Huttenlocher PR, de Courten C, Garey LJ, Van der Loos H (1982). Synaptogenesis in human visual cortex--evidence for synapse elimination during normal development. Neurosci Lett, 33:247-252. 7162689 10.1016/0304-3940(82)90379-2

[28] Sanchez RM, Jensen FE (2001). Maturational aspects of epilepsy mechanisms and consequences for the immature brain. Epilepsia, 42:577-585. 11380563 10.1046/j.1528-1157.2001.12000.x

[29] Sánchez Fernández I, Loddenkemper T (2014). Subunit Composition of Neurotransmitter Receptors in the Immature and in the Epileptic Brain. Biomed Res Int. doi: 10.1155/2014/301950. PMC4180637 25295256

[30] Diering GH, Huganir RL (2018). The AMPA Receptor Code of Synaptic Plasticity. Neuron, 100:314-329. 30359599 10.1016/j.neuron.2018.10.018 PMC6214363

[31] Kumar SS, Bacci A, Kharazia V, Huguenard JR (2002). A developmental switch of AMPA receptor subunits in neocortical pyramidal neurons. J Neurosci, 22:3005-3015. 11943803 10.1523/JNEUROSCI.22-08-03005.2002 PMC6757523

[32] Sanchez RM, Koh S, Rio C, Wang C, Lamperti ED, Sharma D, et al (2001). Decreased glutamate receptor 2 expression and enhanced epileptogenesis in immature rat hippocampus after perinatal hypoxia-induced seizures. J Neurosci, 21:8154-8163. 11588188 10.1523/JNEUROSCI.21-20-08154.2001 PMC6763879

[33] Klein P, Dingledine R, Aronica E, Bernard C, Bl++mcke I, Boison D, et al (2018). Commonalities in epileptogenic processes from different acute brain insults: Do they translate? Epilepsia, 59:37-66. 29247482 10.1111/epi.13965 PMC5993212

[34] Goldberg EM, Coulter DA (2013). Mechanisms of epileptogenesis: A convergence on neural circuit dysfunction. Nature Reviews Neuroscience, 14:337-349. 23595016 10.1038/nrn3482 PMC3982383

[35] Dingledine R, Varvel NH, Dudek FE (2014). When and How Do Seizures Kill Neurons, and Is Cell Death Relevant to Epileptogenesis? Adv Exp Med Biol, 813:109-122. 25012371 10.1007/978-94-017-8914-1_9 PMC4624106

[36] Buckmaster PS, Dudek FE (1997). Neuron loss, granule cell axon reorganization, and functional changes in the dentate gyrus of epileptic kainate-treated rats. J Comp Neurol, 385:385-404. 9300766

[37] Kobayashi M, Buckmaster PS (2003). Reduced Inhibition of Dentate Granule Cells in a Model of Temporal Lobe Epilepsy. J Neurosci, 23:2440-2452. 12657704 10.1523/JNEUROSCI.23-06-02440.2003 PMC6741996

[38] Shao L-R, Dudek FE (2011). Repetitive Perforant-Path Stimulation Induces Epileptiform Bursts in Minislices of Dentate Gyrus From Rats With Kainate-Induced Epilepsy. J Neurophysiol, 105:522-527. 21148094 10.1152/jn.00456.2010 PMC3059160

[39] Maglóczky Z, Freund TF (2005). Impaired and repaired inhibitory circuits in the epileptic human hippocampus. Trends Neurosci, 28:334-340. 15927690 10.1016/j.tins.2005.04.002

[40] Burman RJ, Selfe JS, Lee JH, van den Berg M, Calin A, Codadu NK, et al (2019). Excitatory GABAergic signalling is associated with benzodiazepine resistance in status epilepticus. Brain, 142:3482-3501. 31553050 10.1093/brain/awz283 PMC6904319

[41] Scharfman HE, Brooks-Kayal AR (2014). Is Plasticity of GABAergic Mechanisms Relavant to Epileptogenesis? Adv Exp Med Biol, 813:133-150. 25012373 10.1007/978-94-017-8914-1_11 PMC4370216

[42] Nardou R, Ferrari DC, Ben-Ari Y (2013). Mechanisms and effects of seizures in the immature brain. Semin Fetal Neonatal Med, 18:175-184. 23702158 10.1016/j.siny.2013.02.003

[43] Hyde TM, Lipska BK, Ali T, Mathew SV, Law AJ, Metitiri OE, et al (2011). Expression of GABA Signaling Molecules KCC2, NKCC1, and GAD1 in Cortical Development and Schizophrenia. J Neurosci, 31:11088-11095. 21795557 10.1523/JNEUROSCI.1234-11.2011 PMC3758549

[44] Kipnis PA, Sullivan BJ, Carter BM, Kadam SD (2020). TrkB agonists prevent postischemic emergence of refractory neonatal seizures in mice. JCI Insight. doi: 10.1172/jci.insight.136007. PMC7406252 32427585

[45] Sullivan BJ, Kipnis PA, Carter BM, Kadam SD (2020). Targeting ischemia-induced KCC2 hypofunction rescues refractory neonatal seizures and mitigates epileptogenesis in a mouse model. bioRxiv, 2020.09.15.298596. 10.1126/scisignal.abg2648 PMC8763016 34752143

[46] Cutler CP, Cramb G (2002). Two isoforms of the Na+/K+/2Cl- cotransporter are expressed in the European eel (Anguilla anguilla). Biochim Biophys Acta, 1566:92-103. 12421541 10.1016/s0005-2736(02)00596-5

[47] Begum G, Yuan H, Kahle KT, Li L, Wang S, Shi Y, et al (2015). Inhibition of WNK3 Kinase Signaling Reduces Brain Damage and Accelerates Neurological Recovery After Stroke. Stroke, 46:1956-1965. 26069258 10.1161/STROKEAHA.115.008939 PMC4643659

[48] Kahle KT, Schmouth JF, Lavastre V, Latremoliere A, Zhang J, Andrews N, et al (2016). Inhibition of the kinase WNK1/HSN2 ameliorates neuropathic pain by restoring GABA inhibition. SciSignal, 9:ra32-ra32. 10.1126/scisignal.aad0163 PMC5723157 27025876

[49] Kharod SC, Kang SK, Kadam SD (2019). Off-label use of bumetanide for brain disorders: An overview. Front Neurosci. doi: 10.3389/fnins.2019.00310. PMC6491514 31068771

[50] Flagella M, Clarke LL, Miller ML, Erway LC, Giannella RA, Andringa A, et al (1999). Mice lacking the basolateral Na-K-2Cl cotransporter have impaired epithelial chloride secretion and are profoundly deaf. J Biol Chem, 274:26946-26955. 10480906 10.1074/jbc.274.38.26946

[51] Stödberg T, Magnusson M, Lesko N, Wredenberg A, Martin Munoz D, Stranneheim H, et al (2020). SLC12A2 mutations cause NKCC1 deficiency with encephalopathy and impaired secretory epithelia. Neurol Genet, 6:e478. 32754646 10.1212/NXG.0000000000000478 PMC7357422

[52] Sedmak G, Jovanov-Milošević N, Puskarjov M, Ulamec M, Krušlin B, Kaila K, et al (2016). Developmental Expression Patterns of KCC2 and Functionally Associated Molecules in the Human Brain. Cereb Cortex, 26:4574-4589. 26428952 10.1093/cercor/bhv218

[53] Kaila K, Price TJ, Payne JA, Puskarjov M, Voipio J (2014). Cation-chloride cotransporters in neuronal development, plasticity and disease. Nat Rev Neurosci, 15:637-654. 25234263 10.1038/nrn3819 PMC4294553

[54] Markkanen M, Karhunen T, Llano O, Ludwig A, Rivera C, Uvarov P, et al (2014). Distribution of neuronal KCC2a and KCC2b isoforms in mouse CNS. J Comp Neurol, 522:1897-1914. 24639001 10.1002/cne.23510

[55] Dubois CJ, Cardoit L, Schwarz V, Markkanen M, Airaksinen MS, Uvarov P, et al (2018). Role of the K+-Cl- Cotransporter KCC2a Isoform in Mammalian Respiration at Birth. eNeuro. doi: 10.1523/ENEURO.0264-18.2018. PMC6220586 30406192

[56] Kahle KT, Deeb TZ, Puskarjov M, Silayeva L, Liang B, Kaila K, et al (2013). Modulation of neuronal activity by phosphorylation of the K-Cl cotransporter KCC2. Trends Neurosci, 36:726-737. 24139641 10.1016/j.tins.2013.08.006 PMC4381966

[57] Lee HHC, Walker JA, Williams JR, Goodier RJ, Payne JA, Moss SJ (2007). Direct Protein Kinase C-dependent Phosphorylation Regulates the Cell Surface Stability and Activity of the Potassium Chloride Cotransporter KCC2. J Biol Chem, 282:29777-29784. 17693402 10.1074/jbc.M705053200

[58] Lee HHC, Deeb TZ, Walker JA, Davies PA, Moss SJ (2011). NMDA receptor activity downregulates KCC2 resulting in depolarizing GABA_A_ receptor-mediated currents. Nature Neuroscience, 14:736-743. 21532577 10.1038/nn.2806 PMC3102766

[59] de Los Heros P, Alessi DR, Gourlay R, Campbell DG, Deak M, Macartney TJ, et al (2014). The WNK-regulated SPAK/OSR1 kinases directly phosphorylate and inhibit the K+-Cl- co-transporters. Biochem J, 458:559-573. 24393035 10.1042/BJ20131478 PMC3940040

[60] Moore YE, Deeb TZ, Chadchankar H, Brandon NJ, Moss SJ (2018). Potentiating KCC2 activity is sufficient to limit the onset and severity of seizures. PNAS, 115:10166-10171. 30224498 10.1073/pnas.1810134115 PMC6176565

[61] Hubner CA, Stein V, Hermans-Borgmeyer I, Meyer T, Ballanyi K, Jentsch TJ (2001). Disruption of KCC2 Reveals an Essential Role of K-Cl Cotransport Already in Early Synaptic Inhibition. Neuron, 30:515-524. 11395011 10.1016/s0896-6273(01)00297-5

[62] Li H, Khirug S, Cai C, Ludwig A, Blaesse P, Kolikova J, et al (2007). KCC2 interacts with the dendritic cytoskeleton to promote spine development. Neuron, 56:1019-1033. 18093524 10.1016/j.neuron.2007.10.039

[63] Ben-Ari Y (2017). NKCC1 Chloride Importer Antagonists Attenuate Many Neurological and Psychiatric Disorders. Trends in Neurosciences, 40:536-554. 28818303 10.1016/j.tins.2017.07.001

[64] Penzes P, Cahill ME, Jones KA, VanLeeuwen JE, Woolfrey KM (2011). Dendritic spine pathology in neuropsychiatric disorders. Nat Neurosci, 14:285-293. 21346746 10.1038/nn.2741 PMC3530413

[65] Wong M, Guo D (2013). Dendritic spine pathology in epilepsy: Cause or consequence? Neuroscience, 251:141-150. 22522469 10.1016/j.neuroscience.2012.03.048

[66] Kharod SC, Carter BM, Kadam SD (2018). Pharmaco-resistant Neonatal Seizures: Critical Mechanistic Insights from a Chemoconvulsant Model. Dev Neurobiol, 78:1117-1130. 30136373 10.1002/dneu.22634 PMC6214781

[67] Aguado F (2003). BDNF regulates spontaneous correlated activity at early developmental stages by increasing synaptogenesis and expression of the K+/Cl- co-transporter KCC2. Development, 130:1267-1280. 12588844 10.1242/dev.00351

[68] Kang S, Kadam S (2014). Pre-Clinical Models of Acquired Neonatal Seizures: Differential Effects of Injury on Function of Chloride Co-Transporters. Austin J Cerebrovasc Dis Stroke 1:. PMC4290373 25590049

[69] Carter BM, Sullivan BJ, Landers JR, Kadam SD (2018). Dose-dependent reversal of KCC2 hypofunction and phenobarbital-resistant neonatal seizures by ANA12. Scientific Reports, 8:11987. 30097625 10.1038/s41598-018-30486-7 PMC6086916

[70] Huberfeld G (2007). Perturbed chloride homeostasis and GABAergic signaling in human temporal lobe epilepsy. J Neurosci, 27:9866-9873. 17855601 10.1523/JNEUROSCI.2761-07.2007 PMC6672644

[71] Gharaylou Z, Oghabian MA, Azizi Z, Hadjighassem M (2019). Brain microstructural abnormalities correlate with KCC2 downregulation in refractory epilepsy. Neuroreport, 30:409-414. 30817684 10.1097/WNR.0000000000001216

[72] Hadjikhani N, Asberg Johnels J, Lassalle A, Zurcher NR, Hippolyte L, Gillberg C, et al (2018). Bumetanide for autism: more eye contact, less amygdala activation. Scientific Reports, 8:3602. 29483603 10.1038/s41598-018-21958-x PMC5827728

[73] Greenfield LJ (2013). Molecular Mechanisms of Antiseizure Drug Activity at GABAA Receptors. Seizure, 22:589-600. 23683707 10.1016/j.seizure.2013.04.015 PMC3766376

[74] Boylan GB, Pressler RM (2013). Neonatal seizures: the journey so far. SeminFetal Neonatal Med, 18:173-174. 10.1016/j.siny.2013.05.011 23790527

[75] Sullivan BJ, Kadam SD (2020). The involvement of neuronal chloride transporter deficiencies in epilepsy. In: Tang X, editor. Neuronal Chloride Transporters in Health and Disease. Academic Press, 329-366.

[76] Romermann K, Fedrowitz M, Hampel P, Kaczmarek E, Tollner K, ETraynelis SF, et al (2017). Multiple blood-brain barrier transport mechanisms limit bumetanide accumulation, and therapeutic potential, in the mammalian brain. Neuropharmacology, 117:182-194. 28192112 10.1016/j.neuropharm.2017.02.006

[77] Kang SK, Markowitz GJ, Kim ST, Johnston MV, Kadam SD (2015). Age- and sex-dependent susceptibility to phenobarbital-resistant neonatal seizures: role of chloride co-transporters. Frontiers in Cellular Neuroscience, 9:173. 26029047 10.3389/fncel.2015.00173 PMC4429249

[78] Johne M, Römermann K, Hampel P, Gailus B, Theilmann W, Ala-Kurikka T, et al (2020). Phenobarbital and midazolam suppress neonatal seizures in a noninvasive rat model of birth asphyxia, whereas bumetanide is ineffective. Epilepsia. doi: 10.1111/epi.16778. 33258158

[79] Mazarati A, Shin D, Sankar R (2009). Bumetanide inhibits rapid kindling in neonatal rats. Epilepsia, 50:2117-2122. 19260939 10.1111/j.1528-1167.2009.02048.x PMC2732750

[80] NCT01434225 (2015). NEMO1:NEonatal Seizure Using Medication Off-patent(NEMO1). 2015.

[81] Soul JS, Bergin AM, Stopp C, Hayes B, Singh A, Fortuno CR, et al (2020). A pilot randomized, controlled, double-blind trial of bumetanide to treat neonatal seizures Controlled bumetanide trial for neonatal seizures. Ann Neurol. doi: 10.1002/ana.25959. PMC8122513 33201535

[82] Lemonnier E, Degrez C, Phelep M, Tyzio R, Josse F, Grandgeorge M, et al (2012). A randomised controlled trial of bumetanide in the treatment of autism in children. Translational Psychiatry, 2:e202. 23233021 10.1038/tp.2012.124 PMC3565189

[83] Lemonnier E, Villeneuve N, Sonie S, Serret S, Rosier A, Roue M, et al (2017). Effects of bumetanide on neurobehavioral function in children and adolescents with autism spectrum disorders. Translational Psychiatry, 7:e1056. 28291262 10.1038/tp.2017.10 PMC5416661

[84] Du L, Shan L, Wang B, Li H, Xu Z, Staal WG, et al (2015). A Pilot Study on the Combination of Applied Behavior Analysis and Bumetanide Treatment for Children with Autism. Journal of Child and Adolescent Psychopharmacology, 25:585-588. 26258842 10.1089/cap.2015.0045

[85] Hajri M, Ben Amor A, Abbes Z, Dhouib S, Ouanes S, Mrabet A, et al (2019). Bumetanide in the management of autism. Tunisian experience in Razi Hospital. Tunis Med, 97:971-977. 32173844

[86] van Andel DM, Sprengers JJ, Oranje B, Scheepers FE, Jansen FE, Bruining H (2020). Effects of bumetanide on neurodevelopmental impairments in patients with tuberous sclerosis complex: an open-label pilot study. Molecular Autism, 11:30. 32381101 10.1186/s13229-020-00335-4 PMC7204231

[87] Zhang L, Huang CC, Dai Y, Luo Q, Ji Y, Wang K, et al (2020). Symptom improvement in children with autism spectrum disorder following bumetanide administration is associated with decreased GABA/glutamate ratios. Translational Psychiatry, 10:1-12. 32066666 10.1038/s41398-020-0692-2 PMC7026137

[88] Vlaskamp C, Poil SS, Jansen F, Linkenkaer-Hansen K, Durston S, Oranje B, et al (2017). Bumetanide As a Candidate Treatment for Behavioral Problems in Tuberous Sclerosis Complex. Frontiers in Neurology, 8:469. 28943860 10.3389/fneur.2017.00469 PMC5596068

[89] Tollner K, Brandt C, Topfer M, Brunhofer G, Erker T, Gabriel M, et al (2014). A novel prodrug-based strategy to increase effects of bumetanide in epilepsy. Ann Neurol, 75:550-562. 24615913 10.1002/ana.24124

[90] Lykke K, Tollner K, Feit PW, Erker T, MacAulay N, Loscher W (2016). The search for NKCC1-selective drugs for the treatment of epilepsy: Structure-function relationship of bumetanide and various bumetanide derivatives in inhibiting the human cation-chloride cotransporter NKCC1A. Epilepsy & Behavior, 59:42-49. 27088517 10.1016/j.yebeh.2016.03.021

[91] Romijn HJ, Hofman MA, Gramsbergen A (1991). At what age is the developing cerebral cortex of the rat comparable to that of the full-term newborn human baby? Early Hum Dev, 26:61-67. 1914989 10.1016/0378-3782(91)90044-4

[92] Auvin S, Charriaut-Marlangue C (2017). Role of seizure in neonatal stroke. Oncotarget, 8:48531-48532. 28562317 10.18632/oncotarget.18212 PMC5564704

[93] Bejot Y, Prigent-Tessier A, Cachia C, Giroud M, Mossiat C, Bertrand N, et al (2011). Time-dependent contribution of non neuronal cells to BDNF production after ischemic stroke in rats. Neurochemistry International, 58:102-111. 21074587 10.1016/j.neuint.2010.10.019

[94] Medina I, Friedel P, Rivera C, Kahle KT, Kourdougli N, Uvarov P, et al (2014). Current view on the functional regulation of the neuronal K+-Cl- cotransporter KCC2. Front Cell Neurosci. doi: 10.3389/fncel.2014.00027. PMC3915100 24567703

[95] Lai MC, Yang SN (2011). Perinatal Hypoxic-Ischemic Encephalopathy. J Biomed Biotechnol. 2011: 609813 21197402 10.1155/2011/609813 PMC3010686

[96] Kokaia Z, Andsberg G, Yan Q, Lindvall O (1998). Rapid Alterations of BDNF Protein Levels in the Rat Brain after Focal Ischemia: Evidence for Increased Synthesis and Anterograde Axonal Transport. Experimental Neurology, 154:289-301. 9878168 10.1006/exnr.1998.6888

[97] Rivera C, Li H, Thomas-Crusells J, Lahtinen H, Viitanen T, Nanobashvili A, et al (2002). BDNF-induced TrkB activation down-regulates the K+-Cl- cotransporter KCC2 and impairs neuronal Cl- extrusion. J Cell Biol, 159:747-752. 12473684 10.1083/jcb.200209011 PMC2173387

[98] Cazorla M, Prémont J, Mann A, Girard N, Kellendonk C, Rognan D (2011). Identification of a low-molecular weight TrkB antagonist with anxiolytic and antidepressant activity in mice. J Clin Invest, 121:1846-1857. 21505263 10.1172/JCI43992 PMC3083767

[99] Feng Y, Yang H, Yue Y, Tian F (2020). MicroRNAs and target genes in epileptogenesis. Epilepsia, 61:2086-2096. 32944964 10.1111/epi.16687

[100] Faraoni I, Antonetti FR, Cardone J, Bonmassar E (2009). miR-155 gene: a typical multifunctional microRNA. Biochim Biophys Acta, 1792:497-505. 19268705 10.1016/j.bbadis.2009.02.013

[101] Varendi K, Kumar A, Härma M-A, Andressoo J-O (2014). miR-1, miR-10b, miR-155, and miR-191 are novel regulators of BDNF. Cell Mol Life Sci, 71:4443-4456. 24804980 10.1007/s00018-014-1628-x PMC4207943

[102] Holmes GL, Gairsa J-L, Chevassus-Au-Louis N, Ben-Ari Y (1998). Consequences of neonatal seizures in the rat: Morphological and behavioral effects. Annals of Neurology, 44:845-857. 9851428 10.1002/ana.410440602

[103] Diaz-Arrastia R, Agostini MA, Frol AB, Mickey B, Fleckenstein J, Bigio E, et al (2000). Neurophysiologic and neuroradiologic features of intractable epilepsy after traumatic brain injury in adults. Arch Neurol, 57:1611-1616. 11074793 10.1001/archneur.57.11.1611

[104] Swartz BE, Houser CR, Tomiyasu U, Walsh GO, DeSalles A, Rich JR, et al (2006). Hippocampal cell loss in posttraumatic human epilepsy. Epilepsia, 47:1373-1382. 16922884 10.1111/j.1528-1167.2006.00602.x

[105] Pitkänen A, Immonen R (2014). Epilepsy Related to Traumatic Brain Injury. Neurotherapeutics, 11:286-296. 24554454 10.1007/s13311-014-0260-7 PMC3996118

[106] Löscher W, Hirsch LJ, Schmidt D (2015). The enigma of the latent period in the development of symptomatic acquired epilepsy - Traditional view versus new concepts. Epilepsy Behav, 52:78-92. 26409135 10.1016/j.yebeh.2015.08.037

[107] Chang EF, Englot DJ, Vadera S (2015). Minimally invasive surgical approaches for temporal lobe epilepsy. Epilepsy Behav, 47:24-33. 26017774 10.1016/j.yebeh.2015.04.033 PMC4814159

[108] Helmstaedter C, Kurthen M, Lux S, Reuber M, Elger CE (2003). Chronic epilepsy and cognition: a longitudinal study in temporal lobe epilepsy. Ann Neurol, 54:425-432. 14520652 10.1002/ana.10692

[109] Palma E, Amici M, Sobrero F, Spinelli G, Di Angelantonio S, Ragozzino D, et al (2006). Anomalous levels of Cl- transporters in the hippocampal subiculum from temporal lobe epilepsy patients make GABA excitatory. Proc Natl Acad Sci USA, 103:8465-8468. 16709666 10.1073/pnas.0602979103 PMC1482515

[110] Cohen I, Navarro V, Clemenceau S, Baulac M, Miles R (2002). On the Origin of Interictal Activity in Human Temporal Lobe Epilepsy in Vitro. Science, 298:1418. 12434059 10.1126/science.1076510

[111] Liu G, Gu B, He X-P, Joshi RB, Wackerle HD, Rodriguiz RM, et al (2013). Transient Inhibition of TrkB Kinase Following Status Epilepticus Prevents Development of Temporal Lobe Epilepsy. Neuron, 79:31-38. 23790754 10.1016/j.neuron.2013.04.027 PMC3744583

[112] Huang YZ, He X-P, Krishnamurthy K, McNamara JO (2019). TrkB-Shc Signaling Protects against Hippocampal Injury Following Status Epilepticus. J Neurosci, 39:4624-4630. 30926745 10.1523/JNEUROSCI.2939-18.2019 PMC6554629

[113] Patterson KP, Baram TZ, Shinnar S (2014). Origins of Temporal Lobe Epilepsy: Febrile Seizures and Febrile Status Epilepticus. Neurotherapeutics, 11:242-250. 24604424 10.1007/s13311-014-0263-4 PMC3996115

[114] Shinnar R, Shinnar S, Hesdorffer DC, O’Hara K, Conklin T, Cornett K, et al (2017). Parental Stress, Pediatric Quality of Life and Behavior at Baseline and One Year Follow-up: Results from the FEBSTAT Study. Epilepsy Behav, 69:95-99. 28236729 10.1016/j.yebeh.2017.01.024 PMC5423815

[115] Puskarjov M, Seja P, Heron SE, Williams TC, Ahmad F, Iona X, et al (2014). A variant of KCC2 from patients with febrile seizures impairs neuronal Cl- extrusion and dendritic spine formation. EMBO Rep, 15:723-729. 24668262 10.1002/embr.201438749 PMC4197883

[116] Malmgren K, Thom M (2012). Hippocampal sclerosis--origins and imaging. Epilepsia, 53 Suppl 4:19-33. 22946718 10.1111/j.1528-1167.2012.03610.x

[117] Cavarsan CF, Malheiros J, Hamani C, Najm I, Covolan L (2018). Is Mossy Fiber Sprouting a Potential Therapeutic Target for Epilepsy? Front Neurol, 9:1023. 30555406 10.3389/fneur.2018.01023 PMC6284045

[118] Muzumdar D, Patil M, Goel A, Ravat S, Sawant N, Shah U (2016). Mesial temporal lobe epilepsy - An overview of surgical techniques. Int J Surg, 36:411-419. 27773861 10.1016/j.ijsu.2016.10.027

[119] Kadam SD, Dudek FE (2007). Neuropathogical features of a rat model for perinatal hypoxic-ischemic encephalopathy with associated epilepsy. J Comp Neurol, 505:716-737. 17948865 10.1002/cne.21533 PMC4607042

[120] Hampel P, Römermann K, Gailus B, Johne M, Gericke B, Kaczmarek E, et al (2021). Effects of the NKCC1 inhibitors bumetanide, azosemide, and torasemide alone or in combination with phenobarbital on seizure threshold in epileptic and nonepileptic mice. Neuropharmacology, 185:108449. 33450274 10.1016/j.neuropharm.2021.108449

[121] Baud MO, Kleen JK, Mirro EA, Andrechak JC, King-Stephens D, Chang EF, et al (2018). Multi-day rhythms modulate seizure risk in epilepsy. Nature Communications, 9:88. 10.1038/s41467-017-02577-y PMC5758806 29311566

[122] Ulate-Campos A, Tsuboyama M, Loddenkemper T (2017). Devices for Ambulatory Monitoring of Sleep-Associated Disorders in Children with Neurological Diseases. Children (Basel). doi: 10.3390/children5010003. PMC5789285 29295578

[123] Helmstaedter C, Elger CE (2009). Chronic temporal lobe epilepsy: a neurodevelopmental or progressively dementing disease? Brain, 132:2822-2830. 19635728 10.1093/brain/awp182

[124] Boylan GB, Stevenson NJ, Vanhatalo S (2013). Monitoring neonatal seizures. SeminFetal Neonatal Med, 18:202-208. 10.1016/j.siny.2013.04.004 23707519

